# Risky working conditions and chronic kidney disease

**DOI:** 10.1186/s12995-023-00393-3

**Published:** 2023-11-14

**Authors:** Rui Lan, Yao Qin, Xiangjun Chen, Jinbo Hu, Wenjin Luo, Yan Shen, Xue Li, Lina Mao, Hanwen Ye, Zhihong Wang

**Affiliations:** Department of Endocrinology, The First Affiliated Hospital of Chongqing Medical University, No.1 Youyi Street, Yuzhong District, Chongqing, 400016 China

**Keywords:** Occupational, Chronic kidney disease, Work environments

## Abstract

**Background:**

Individuals in the workplace are exposed to various environments, tasks, and schedules. Previous studies have indicated a link between occupational exposures and an increased risk of chronic kidney disease (CKD). However, the social conditions of the work environment may also be a crucial contributing factor to CKD. Furthermore, individuals may encounter multiple occupational-related risk factors simultaneously, underscoring the importance of investigating the joint risk of different working conditions on CKD.

**Methods:**

A prospective analysis of 65,069 UK Biobank participants aged 40 to 69 years without CKD at baseline (2006–2010) was performed. A self-administered questionnaire assessed working conditions and a working conditions risk score were developed. Participants who answered “sometimes” or “often” exposure to occupational heat or occupational secondhand cigarette smoke; involved in shift work or heavy workloads (“usually” or “always”), were grouped as high-risk working conditions. Each working condition was scored as 1 if grouped as high-risk, and 0 if not. The working conditions risk score was equal to the sum of these four working conditions. Cox proportional hazard regression models were used to estimate the associations between working conditions and CKD incidence.

**Results:**

The mean follow-up time was 6.7 years. After adjusting for demographic, lifestyle, and working time factors, the hazard ratios for the development of CKD for heavy workloads, shift work, occupational secondhand cigarette smoke exposure, and occupational heat exposure were 1.24 (95%CI = 1.03, 1.51), 1.33 (95%CI = 1.10, 1.62), 1.13 (95%CI = 1.01, 1.26), 1.11 (95%CI = 0.99, 1.24), respectively. The risk of CKD was found to be significantly associated with an increasing working conditions risk score. Individuals with a working conditions risk score of 4 had an 88.0% (95% CI = 1.05, 3.35) higher risk of developing CKD when compared to those with a working conditions risk score of 0.

**Conclusions:**

Adverse working conditions, particularly when considered in combination, can significantly elevate the risk of chronic kidney disease (CKD). These results provide a reference for implementing measures to prevent CKD.

**Supplementary Information:**

The online version contains supplementary material available at 10.1186/s12995-023-00393-3.

## Introduction

The prevalence of chronic kidney disease (CKD) has been high for a long time. Worsely, the risk of death caused by CKD has increased year by year, which has brought a huge burden on the global economy and life expectancy [1, 2]. CKD has already become one of the public health problems that have attracted wide attention all over the world.

The development of CKD is strongly associated with occupational and environmental factors [3]. Previous studies confirmed that risky working conditions, like heavy workloads, long working hours, and shift work are risk factors for diabetes, cardiovascular disease, and death [4–7]. The risky work conditions are also considered associated with kidney function [8–10]. For example, a growing number of manual workers in hot, agricultural communities were reported to suffer from CKD [3]. In addition, many people are often exposed to secondhand cigarette smoke in workplace. Previous studies also found that secondhand cigarette smoke exposure is significantly associated with the development of CKD [11–13]. To our knowledge, most of these risky working conditions were assessed cross-sectionally or individually without taking into account the complexity and correlations of various work conditions. The joint risk of various working conditions, especially heat exposure and heavy workloads, on CKD needs to be explored.

In this prospective study, we investigated the relationship between individual working conditions, including heavy workloads, shift work, occupational secondhand cigarette smoke exposure, and occupational heat exposure, and the risk of developing CKD. Then the working condition risk score was developed based on the above working conditions, and its association with CKD was also estimated.

## Methods

### Study population

**Fig. 1:**
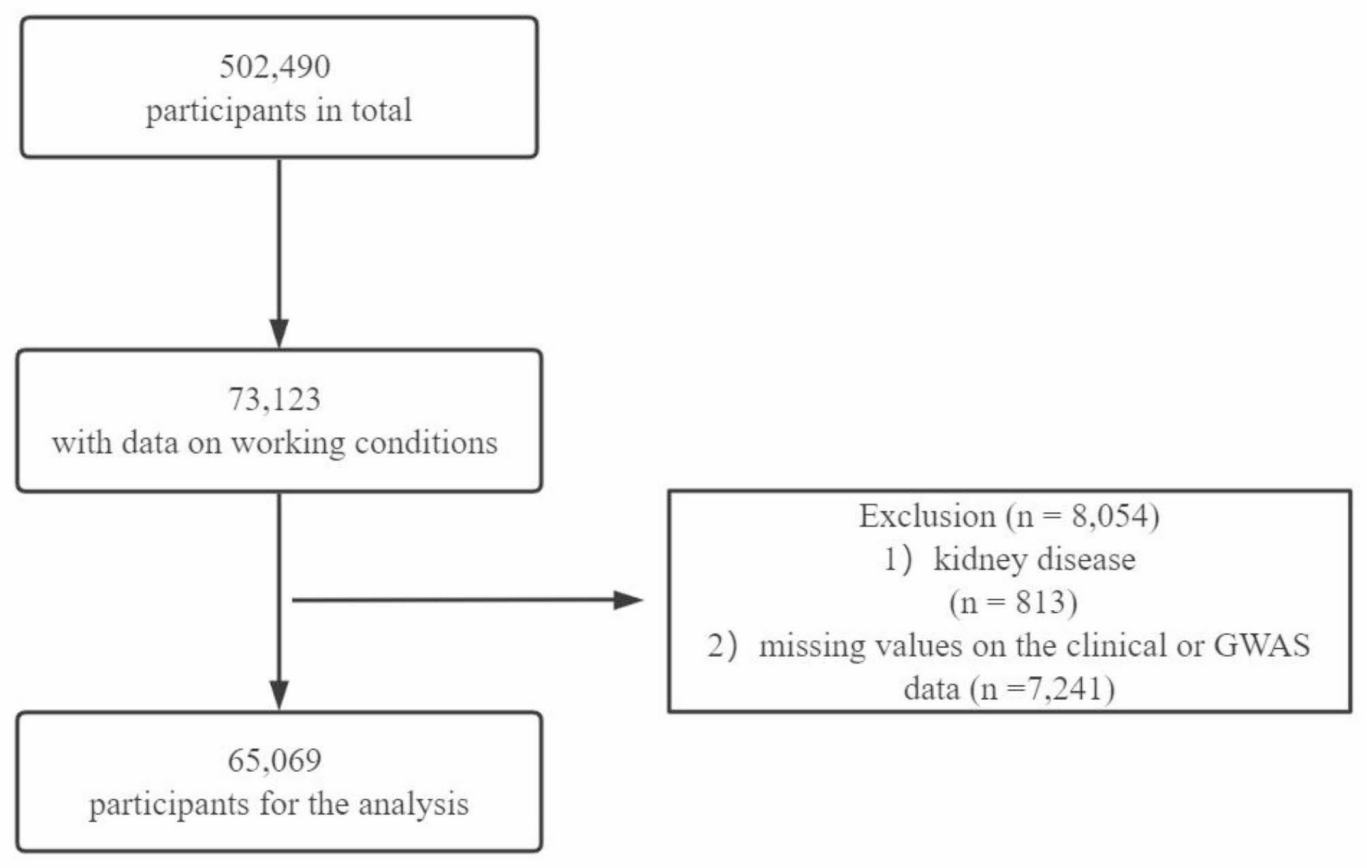
Flow chart of the study population. GWAS: genome-wide association

The UK Biobank is a large prospective information resource that recruited more than half a million people aged 40–69 years (2006 to 2010) and collected their genetic information, blood samples, lifestyle, and environmental exposure data. The relevant information has been described in detail elsewhere [14, 15]. The current analysis was approved by UK Biobank with an ID of 66,536. All patients signed informed consent at the time of the first enrollment. This analysis received approval from the National Information Governance Board for Health and Social Care and the National Health Service North West Multicentre Research Ethics Committee (reference 13/NW/0382). At baseline, we included individuals with complete data on the aforementioned four working conditions (n = 73,123). Subsequently, we excluded participants with CKD (n = 813) and those with missing values in the clinical data (n = 7,241), resulting in a final analysis cohort of 65,069 participants (Fig. 1).

### Measures

The primary outcome in the present study was incident CKD. CKD was defined according to the International Classification of Diseases-10th Edition, including diabetes mellitus with renal complications (E10.2, E11.2, E12.2, E13.2, and E14.2), hypertensive renal disease (I12 and I13), glomerular disease (N03, N04, N05, and N07), renal tubulointerstitial disease (N11, N12, N13, N14, and N15), and renal failure (N18 and N19). In the UK Biobank study, the recorded outcomes and endpoints for each participant were primarily determined by healthcare providers, who utilized a combination of hospital admissions, self-reported information, and death registration data to diagnose CKD, following the definitions provided by ICD-10 codes.

All participants collected information on age, sex, weight, smoking status, alcohol consumption, activity_MET, medication use, and physician diagnosis of chronic diseases via a touchscreen device at baseline. Biospecimens were collected and assayed in the central laboratory. UK Biobank collected the genetic information, we selected a total of 241 independent single nucleotide variations which were identified from the most recent genome-wide association study (GWAS) and were significantly associated with CKD, details information on the selected SNP is provided in Table S1 [16]. The genetic risk score (GRS) for CKD was calculated by a method that has been described elsewhere: GRS = (β1 X SNP1 + β2 X SNP2 +… + β241 X SNP241), each SNP was coded as 0, 1, and 2 according to the number of risk alleles. The β coefficient was obtained from the reported GWAS meta-analysis [17].

Participants were asked the following questions about their jobs: occupational heat exposure was assessed by the question, “Thinking about the place where you worked: Was it very hot?” with multiple choices provided: (1) never/rarely; (2) sometimes; and (3) often. Occupational secondhand cigarette smoke exposure was obtained by the question, “Thinking about the place where you worked: Was there a lot of cigarette smoke from other people smoking?” with multiple choices provided: (1) never/rarely; (2) sometimes; and (3) often. The workload was asked by the question, “Does your work involve heavy manual or physical work?” (Physical work includes work that involves handling heavy objects and use of heavy tools.) with the following choices:1) never/rarely; 2) sometimes; 3) usually; and (4) always. Shift work was assessed by the question “Does your work involve shift work?” (shift work is a work schedule that falls outside of the normal daytime working hours of 9 am-5 pm. This may involve working afternoons, evenings, or nights or rotate through these kinds of shifts) with the following choices:1) never/rarely; 2) sometimes; 3) usually; and 4) always. The working hour was reported as the hours of work time every week (Do not include hours of commute).

Based on the participants’ responses, we obtained their information on the above working conditions. Participants who answered “sometimes” or “often” exposure to occupational heat; reported “sometimes” or “often” exposure to occupational secondhand cigarette smoke; involved in shift work (“usually” or “always”); “usually” or “always” had heavy workloads, were grouped as high-risk working conditions. Each work condition was coded 1 if grouped to high-risk and 0 if not. The working conditions risk score was obtained by summing up the above four work conditions, and higher scores indicate higher levels of risk in working conditions. We also categorized it as healthy working condition (working conditions risk score 0), intermediate working condition (working conditions risk score 1–2), and poor working condition (working conditions risk score 3–4).

### Assessment of other covariates

Demographic and lifestyle behaviors were collected using a touchscreen device. The townsend deprivation index, which is based on the participant’s postcode and reflects the degree of deprivation, was obtained. Physical activity was evaluated by calculating the total metabolic equivalent task (MET) minutes per week. This calculation encompassed minutes spent on all leisure-time activities, including mild activities such as walking, as well as moderate and vigorous activities. Subsequently, the total MET was categorized into tertiles: low, medium, and high. Household income was classified into five levels based on the family’s pre-tax annual income. Education was classified as having a college education or not. Diet was divided into healthy and unhealthy according to a healthy diet score which has been used in several studies successfully, and the healthy diet score was based on the American Heart Association (AHA) guidelines [18–20]. Blood samples were collected and analyzed at the central laboratory.

### Statistical analysis

All of the analyses were performed by R v4.1.2 (http://www.R-project.org, The R 121 Foundation). *P* < 0.05 represents statistical significance. Follow-up time was calculated from the baseline date to diagnosis of CKD, death, or last follow-up date, whichever occurred first. The Cox proportional hazards model was used to calculate the HR and 95% CI between risky working conditions,working conditions risk score, and CKD. Multivariate models were established for controlling potential confounders, including age, sex, ethnic, working time, activity_MET, the townsend deprivation index, smoking status, alcohol consumption, triglyceride, body mass index, hypertension, diabetes mellitus, estimated glomerular filtration rate (eGFR).

To examine the reliability of the results, we additionally adjusted for education, healthy diet, and genetic risk of CKD (GRS_CKD). We further excluded people who had a CKD event within 2 years of follow-up to avoid reverse causality. Calculated the population attributable risk proportion (PAR%) to estimate the proportion of participants who would not theoretically develop CKD if all participants were not exposed to risky working conditions. We also constructed a weighted working conditions risk score based on the 4 work factors by using the equation: weighted work score= (β1×factor1 + β2 ×factor 2 +…+β4×factor 4) × (4/sum of the β coefficients). This weighted score also ranges from 0 to 4 points but considers magnitudes of the adjusted relative risk for each factor in each work pattern as a combination of 4 factors.

## Results

The mean follow-up period was 6.7 years. Of the 65,069 participants, 29.8% had no exposure to high-risk working conditions, while 41.7% of participants had at least one exposure to high-risk working conditions. The numbers of participants who had exposure to one, two, three and four of the risky working conditions were 27,163, 15,305, 2829 and 389, respectively. Participants involved in heavy workloads, shift work, occupational secondhand cigarette smoke exposure, and occupational heat exposure were 6.7%, 12.7%, 45.7% and 44.5%, respectively.

Table S2 shows the baseline characteristics of the study participants grouped according to working conditions risk score. 92% of the total population were white. The average age and body mass index of the total population were 65.6 ± 6.8 years and 26.7 ± 4.5 kg/m^2^, with 45.8% of females, 4.5% of diabetes, and 2.9% of cardiovascular disease. People with higher working conditions risk score were more likely to be female, younger, to work longer hours per week, to have higher baseline eGFR and to have higher proportions of diabetes, hypertension, and cardiovascular disease.

Table 1 shows the relationships between individual working conditions and CKD. After adjusting for age, sex, ethnic, working time, activity_MET, the townsend deprivation index, smoking status, alcohol consumption, triglyceride, body mass index, hypertension, diabetes mellitus and eGFR, the multivariable-adjusted hazard ratios of heavy workloads, shift work, occupational secondhand cigarette smoke exposure, and occupational heat exposure were 1.24 (95%CI = 1.03, 1.51), 1.33 (95%CI = 1.10, 1.62),1.13 (95%CI = 1.01, 1.26), 1.11 (95%CI = 0.99, 1.24) for CKD, compared to the low-risk working conditions, respectively. We also separately calculated PAR% for individual working conditions, which explained 1.8% (heavy workloads) to 6.1% (secondhand smoke) of CKD events.

**Table 1:** Multivariable-adjusted HRs (95% CIs) for CKD by risky working conditions

Risky working conditions	% of 65,069 participants	HR (95% CI)	p-value	PAR%
Heat	44.5	1.11(0.99 ,1.24)	0.069	4.8(-0.4, 10.0)
Shift work	6.7	1.33(1.10, 1.62)	0.004	2.3(0.5, 4.0)
Heavy workloads	7.2	1.24(1.03, 1.51)	0.025	1.8(0.7, 3.5)
Secondhand smoke	45.7	1.13(1.01, 1.26)	0.031	6.1(0.6, 11.6)

Multivariable-adjusted HRs: Age (continuous), Sex (male/female), Ethnic (White/other), Working time(continuous), Activity_MET (low/median/high), The Townsend Deprivation Index (continuous), Smoke status (yes/no), Alcohol consumption (frequence1-6), TG(continuous), Body mass index (continuous), Hypertension (yes/no), DM (yes/no), eGFR(continuous).

Table S3 shows the relationships between individual working conditions and CKD in detail. In the multivariable-adjusted models, compared to who answered “never/rarely” in the question about working conditions, those reported “often” exposed to secondhand smoke in the work environment, “always” involved in shift work, or “always” having heavy workloads were associated with an increased risk of CKD.

When these four working conditions were considered jointly by using working conditions risk score, the risks of CKD increased significantly with an increasing working conditions risk score. (*P* for trend = 0.001), shown in Table 2. Each unit increase in the working conditions risk score was associated with a HR of 1.18 (95%CI = 1.07, 1.30) for CKD. The results were not materially changed for weighted working conditions risk score (Table S4). Compared with participants with a working conditions risk score of 0, those with a working conditions risk score of 4 had an 88.0% (95% CI = 1.05, 3.35) higher risk of developing CKD.

**Table 2:** Multivariable-adjusted HRs (95%CIs) for incident CKD by different working conditions risk scores among 65,069 participants

	Model1		Model2		Model3	
	HR (95% CI)	p-value	HR (95% CI)	p-value	HR (95% CI)	p-value
Work conditions risk score	1.29(1.17, 1.42)	< 0.001	1.24(1.13, 1.37)	< 0.001	1.18(1.07, 1.30)	0.001
0	1.00(reference)	-	1.00(reference)	-	1.00(reference)	-
1	1.21(1.05, 1.39)	0.008	1.20(1.04, 1.38)	0.011	1.14(0.99, 1.32)	0.062
2	1.42(1.22, 1.66)	< 0.001	1.38(1.18, 1.61)	< 0.001	1.25(1.07, 1.46)	0.005
3	1.78(1.38, 2.28)	< 0.001	1.65(1.29, 2.12)	< 0.001	1.47(1.14, 1.88)	0.003
4	2.27(1.28, 4.05)	0.005	2.04(1.15, 3.65)	0.015	1.88(1.05, 3.35)	0.033

Model1. Age (continuous), Sex (male/female), Ethnic (Whiter/other), Working time(continuous). Model2. model1 + Activity_MET (low/median/high), The Townsend Deprivation Index (continuous), Alcohol consumption (frequence1-6), Smoke status (yes/no). Model3. model2 + TG(continuous), Body mass index (continuous), Hypertension (yes/no), DM (yes/no), eGFR(continuous).

**Fig. 2:**
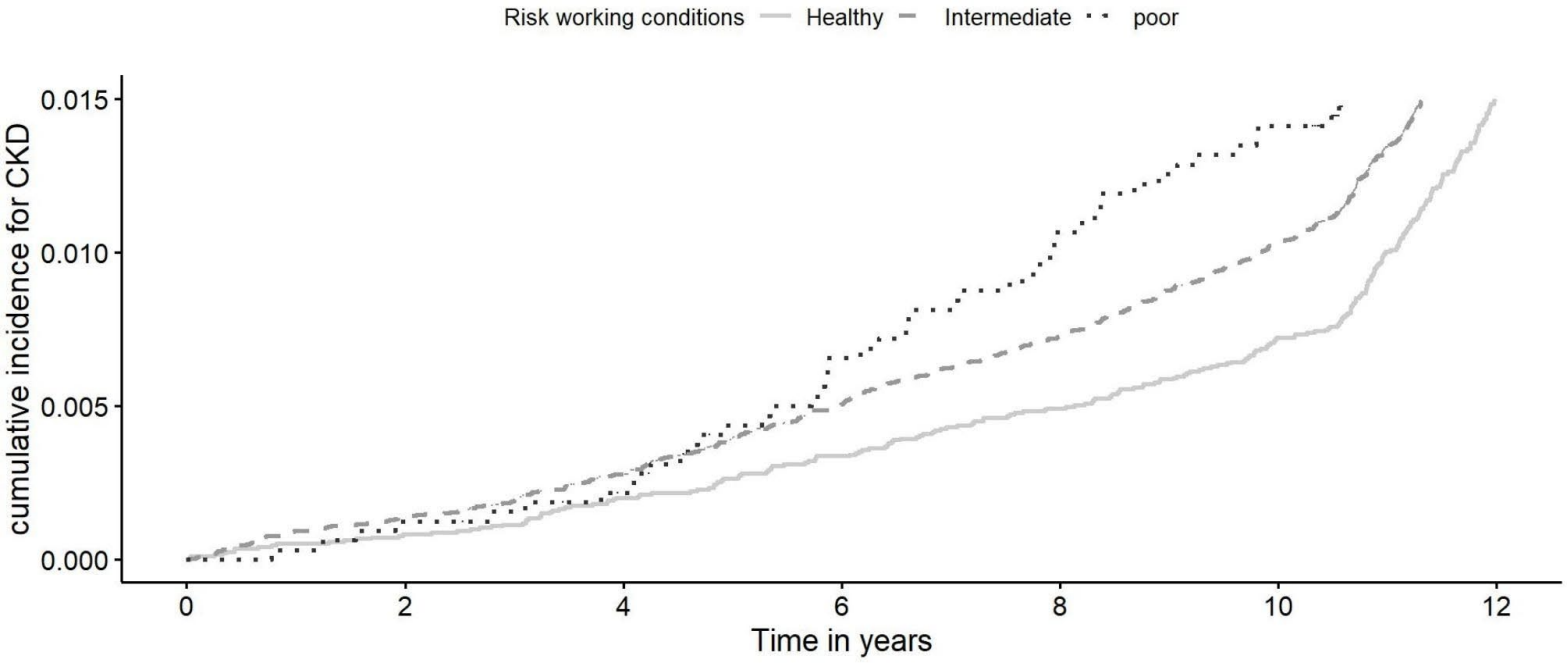
Cumulative incidence for CKD in patients with different working conditions. Working conditions are categorized into 3 groups based on working conditions risk score: healthy working conditions, intermediate working conditions, and poor working conditions. CKD: chronic kidney disease

Table 3 and Fig 2 show the associations between three categories of working conditions risk score (healthy working conditions, intermediate working conditions, and poor working conditions) and the risk of CKD. Compared with the healthy working conditions, participants with poor working conditions were associated with a 50.0% (95%CI = 1.19, 1.91) higher risk of CKD. The associations were slightly attenuated but remained significant after further adjusting for education, healthy diet, and GRS_CKD (Table S5). Excluding incident cases that occurred within the first 2 years of follow up showed similar results, providing evidence against reverse causation (Table S6).

**Table 3:** Multivariable-adjusted HRs (95% CIs) for CKD by the categorized working conditions risk score

	Model1		Model2		Model3	
	HR (95% CI)	p-value	HR (95% CI)	p-value	HR (95% CI)	p-value
Healthy conditions	1.00(reference)	-	1.00(reference)	-	1.00(reference)	-
Intermediate conditions	1.29(1.13, 1.47)	< 0.001	1.27(1.11, 1.44)	< 0.001	1.18(1.04, 1.35)	0.012
Poor conditions	1.82(1.44, 2.31)	< 0.001	1.69(1.33, 2.14)	< 0.001	1.50(1.19, 1.91)	0.001

Model1. Age (continuous), Sex (male/female), Ethnic (Whiter/other), Working time(continuous). Model2. model1 + Activity_MET (low/median/high), The Townsend Deprivation Index (continuous), Alcohol consumption (frequence1-6), Smoke status (yes/no). Model3. model2 + TG(continuous), Body mass index (continuous), Hypertension (yes/no), DM (yes/no), eGFR(continuous).

## Discussion

In this large prospective study, we found that poor working conditions were associated with an increased risk of CKD. Compared to those with a working conditions risk score of 0, subjects with a working conditions risk score of 4 had an 88.0% higher risk of CKD. Adverse working conditions such as heavy workloads, shift work, and occupational secondhand cigarette smoke exposure independently increased the risk of CKD, respectively.

Despite the paucity of high-quality prospective studies, previous studies suggested that shift work was associated with a high prevalence of CKD and a low eGFR [8, 21]. Current results also suggest about 30% of people who are regularly exposed to heat at work (at least six hours a day, five days a week, for two months of the year) will experience kidney disease or acute kidney injury [10, 22, 23]. More importantly, people who have heavy workloads in heat are more likely to develop kidney injury, compared with people with light workloads [24, 25]. Occupational secondhand cigarette smoke exposure has been consistently associated with an increased risk of CKD [11–13]. Notably, a substantial portion of the population is routinely exposed to secondhand cigarette smoke in the workplace, and the impact of such an exposure cannot be underestimated. Therefore, occupational secondhand cigarette smoke exposure also is considered a risky work condition. Previous evidence also observed working time was inversely associated with CKD [9]. Considering that the exposure hours of social environmental factors at work are affected by working time. In our study, working time was adjusted as a confounder. The results of this study showed that heavy workloads, shift work, and occupational secondhand cigarette smoke exposure independently increased the risk of CKD, respectively. Furthermore, our study was conducted in the United Kingdom, which has a temperate maritime climate characterized by relatively low year-round temperatures. This climatic context may have played a role in the lack of significant findings related to occupational heat exposure in our study.

For the first time, our study simultaneously considered a variety of common risky working conditions and constructed the working conditions risk score. Previous studies [8–10, 26], only take a single risky working condition to considered, but workers, such as construction workers and agricultural workers were usually simultaneously encountered more than one adverse factors at the occupational environment. In our study, the joint risk of various working conditions on CKD was assessed. The results showed that participants with poor working condition were associated with an increased risk of CKD compared to those with a relatively healthy working condition. Our finding maybe addressed the previous observed phenomenon that agricultural workers have a higher incidence of unexplained decline in renal function. So it is very important to establish a score of risk working conditions.

Several potential mechanisms could explain the observed associations between the risky working conditions and the increased risk of CKD. Shift work changes circadian rhythm and leads to higher risk of physical and psychological stress [27–30]. It also affects the length and quality of sleep and causes a wide range of metabolic changes, such as blood lipids, glucose metabolism and inflammatory response [6, 31–33]. Heavy workloads and long working hours break good work–life balance, which keeps the psychological and physiological system in a state of tension, leading to enhanced sympathetic nervous system activity, as well as affects the metabolic [7, 27, 34]. Furthermore, individuals engaged in shift work or strenuous physical labor are susceptible to increased sweating and may experience inadequate hydration, potentially leading to dehydration, hyperosmolarity, and elevated serum creatinine levels [3, 35–38]. Secondhand smoke exposure leads to the accumulation of cytotoxic substances and induces inflammatory response, oxidative stress, and activation of the sympathetic system [39, 40]. Various risky working conditions may jointly affect renal function via the same or complementary pathways, therefore the cumulative associations obtained when analyzed jointly various risky working conditions in this study can be explained.

To the best of our knowledge, this is the first large prospective study to investigate the associations between workloads, shift work, occupational heat exposure and the risk of CKD. It is also the first study to establish a score of working risk conditions and examine the relationship between the score and the incidence of CKD. The study highlights the importance of early avoidance or improvement of risky working conditions in the prevention of CKD. The findings support potential interventions aimed at avoiding or improving working conditions to prevent the risk of CKD.

### Limitations

This study has some limitations. Firstly, the evaluation of working conditions relies on patients’ self-reported data at baseline, which introduces an inherently subjective recall bias. To bolster the reliability of our findings, further randomized controlled trials (RCTs) are warranted for verification. Second, the study population was predominantly white, similar investigations in diverse ethnic populations are imperative. Third, although the multi-factors corrected Cox regression method and the results in this study were acceptable and credible, unmeasured or unknown residual confounding is inevitable. Last, the work society and environmental factors involved in this study can not fully summarize all dimensions, such as work environment noise, work environment dust, and so on. Further research is warranted to assess the association between performance in other occupational risk dimensions or specific occupations related to kidney injury and the risk of CKD.

## Conclusions

Adverse working conditions have the potential to increase the risk of CKD. Avoiding or improving working conditions may be an effective way to reduce the incidence of CKD. Future studies are warranted to consider more occupational social environment context in the field of occupational health and preventive medicine.

### Electronic supplementary material

Below is the link to the electronic supplementary material.

Supplementary Material 1

## Data Availability

The access policies and procedures are available at www.ukbiobank.ac.uk.

## Abbreviations

CKD: Chronic kidney disease
GWAS: Genome-wide association study
MET: Metabolic equivalent task
PAR%: Population attributable risk proportion
GRS_CKD: Genetic risk score of CKD
eGFR: Estimated glomerular filtration rate
AHA: American Heart Association
DM: Diabetes mellitus
