# Supplementary material for: Risky working conditions and chronic kidney disease
Source: J Occup Med Toxicol. 2023 Nov 14;18:26. doi: 10.1186/s12995-023-00393-3 (PMC10644450; doi:10.1186/s12995-023-00393-3)
Supplement: Supplementary file 1 — Supplementary Material 1 [file 12995_2023_393_MOESM1_ESM.docx]

Supplementary File 1-1. Components of the genetic risk score by study.

| SNP | CHR | BP | A1 | A2 | OR | P |
| --- | --- | --- | --- | --- | --- | --- |
| rs116626164 | 1 | 50975227 | A | T | 1.1 | 0.0028 |
| rs116760613 | 1 | 51490709 | A | G | 1.09 | 0.0022 |
| rs72661397 | 1 | 52013915 | T | C | 0.93 | 0.0083 |
| rs760077 | 1 | 155178782 | A | T | 1.02 | 0.016 |
| rs2383531 | 1 | 186729401 | A | G | 0.93 | 4.30E-05 |
| rs3791760 | 2 | 10118424 | T | C | 0.98 | 0.013 |
| rs11123170 | 2 | 113978940 | C | G | 0.97 | 1.10E-05 |
| rs1078442 | 2 | 121988924 | A | C | 1.04 | 2.70E-06 |
| rs72929920 | 2 | 177097022 | T | C | 1.14 | 7.50E-07 |
| rs1047891 | 2 | 211540507 | A | C | 1.05 | 3.00E-08 |
| rs2332036 | 3 | 121714391 | T | C | 1.02 | 0.0035 |
| rs28817415 | 4 | 77401452 | T | C | 1.07 | 1.90E-16 |
| rs12509595 | 4 | 81182554 | T | C | 1.07 | 5.10E-14 |
| rs1229984 | 4 | 100239319 | T | C | 1.05 | 0.0025 |
| rs11745300 | 5 | 34504668 | C | G | 0.96 | 3.10E-06 |
| rs4976646 | 5 | 176788570 | T | C | 0.95 | 2.40E-10 |
| rs7766720 | 6 | 107172979 | T | C | 0.97 | 0.0097 |
| rs162185 | 6 | 134226147 | T | C | 0.98 | 0.019 |
| rs13230625 | 7 | 1286244 | A | G | 1.07 | 8.50E-13 |
| rs700753 | 7 | 46753684 | C | G | 0.97 | 0.0011 |
| rs55914958 | 7 | 101237753 | T | C | 0.98 | 0.037 |
| rs73728279 | 7 | 151411494 | T | G | 1.11 | 7.00E-24 |
| rs7834797 | 8 | 23759535 | A | G | 1.04 | 1.20E-06 |
| rs7084402 | 10 | 60265404 | A | G | 1.02 | 0.025 |
| rs10821944 | 10 | 63785089 | T | G | 0.98 | 0.01 |
| rs7096822 | 10 | 126664166 | T | C | 1.02 | 0.011 |
| rs3925584 | 11 | 30760335 | T | C | 1.08 | 2.70E-21 |
| rs11039216 | 11 | 47406592 | T | C | 1.04 | 2.10E-05 |
| rs7123489 | 11 | 65524252 | A | C | 1.06 | 1.60E-11 |
| rs4567493 | 11 | 86634423 | A | G | 1.02 | 0.014 |
| rs7931938 | 11 | 111207105 | A | G | 1.03 | 0.0019 |
| rs7936300 | 11 | 122601034 | A | G | 0.98 | 0.045 |
| rs2122982 | 12 | 57781893 | A | G | 0.96 | 5.40E-05 |
| rs1275609 | 12 | 76271183 | A | G | 0.97 | 0.0012 |
| rs584480 | 13 | 72345505 | T | C | 0.98 | 0.04 |
| rs7327286 | 13 | 73713447 | A | G | 0.97 | 0.0038 |
| rs17730281 | 15 | 53907948 | A | G | 0.92 | 1.40E-18 |
| rs4886755 | 15 | 76298132 | A | G | 0.97 | 2.60E-05 |
| rs12908437 | 15 | 99287375 | T | C | 1.02 | 0.008 |
| rs77924615 | 16 | 20392332 | A | G | 0.81 | 6.70E-86 |
| rs8050136 | 16 | 53816275 | A | C | 1.03 | 2.80E-05 |
| rs6504021 | 17 | 59240473 | T | C | 1.04 | 2.60E-05 |
| rs16942713 | 18 | 24386535 | T | G | 1.07 | 1.00E-09 |
| rs8096658 | 18 | 77156537 | C | G | 0.94 | 2.00E-12 |
| rs7259714 | 19 | 817394 | T | C | 0.98 | 0.0093 |
| rs838144 | 19 | 49250239 | T | C | 0.97 | 0.0027 |
| rs6026580 | 20 | 57468150 | T | C | 0.98 | 0.018 |
| rs2823139 | 21 | 16576783 | A | G | 1.05 | 1.20E-08 |
| rs74748843 | 1 | 10730910 | T | C | 1.06 | 0.0037 |
| rs12061708 | 1 | 18809916 | A | G | 1.03 | 0.00074 |
| rs2749153 | 1 | 23699340 | A | G | 1.04 | 5.00E-06 |
| rs688540 | 1 | 48002447 | A | G | 1.04 | 0.007 |
| rs17413465 | 1 | 55718708 | A | C | 0.98 | 0.04 |
| rs1757915 | 1 | 56615809 | A | G | 0.97 | 8.50E-05 |
| rs679843 | 1 | 78707493 | T | C | 0.98 | 0.039 |
| rs11166440 | 1 | 100808363 | A | G | 0.98 | 0.0045 |
| rs10857788 | 1 | 110012289 | A | G | 0.98 | 0.0078 |
| rs12736457 | 1 | 113258293 | C | G | 0.94 | 2.30E-06 |
| rs267738 | 1 | 150940625 | T | G | 1.05 | 2.10E-05 |
| rs4971100 | 1 | 155155731 | A | G | 0.98 | 0.049 |
| rs3845534 | 1 | 163738950 | A | G | 1.03 | 0.00049 |
| rs4656220 | 1 | 170649277 | T | C | 0.98 | 0.013 |
| rs3850625 | 1 | 201016296 | A | G | 0.97 | 0.024 |
| rs2808454 | 1 | 207231751 | A | T | 0.97 | 5.20E-05 |
| rs75625374 | 1 | 208039431 | C | G | 0.95 | 0.0047 |
| rs417237 | 1 | 228532195 | T | G | 0.98 | 0.0017 |
| rs2490391 | 1 | 243469669 | A | C | 1.07 | 3.70E-17 |
| rs3791221 | 2 | 226933 | A | G | 0.98 | 0.0076 |
| rs807624 | 2 | 15782471 | T | G | 0.98 | 0.0055 |
| rs4491726 | 2 | 18676276 | A | G | 0.98 | 0.022 |
| rs780093 | 2 | 27742603 | T | C | 0.97 | 1.50E-05 |
| rs2301343 | 2 | 40680149 | T | G | 1.02 | 0.044 |
| rs2971880 | 2 | 54885640 | A | T | 1.04 | 5.00E-06 |
| rs6546869 | 2 | 73895765 | A | G | 0.94 | 6.70E-08 |
| rs11123169 | 2 | 113967075 | T | C | 0.95 | 2.30E-10 |
| rs11694902 | 2 | 121988884 | A | G | 0.95 | 2.00E-04 |
| rs7425436 | 2 | 148759656 | A | G | 0.98 | 0.044 |
| rs4664475 | 2 | 152387553 | T | C | 1.03 | 0.0017 |
| rs35472707 | 2 | 169995581 | T | C | 1.09 | 1.60E-05 |
| rs187355703 | 2 | 176993583 | C | G | 0.82 | 2.80E-10 |
| rs35284526 | 2 | 178121524 | A | C | 0.97 | 0.0017 |
| rs4666821 | 2 | 183077254 | T | G | 0.97 | 0.00088 |
| rs60980181 | 2 | 188168567 | A | T | 1.03 | 0.0015 |
| rs1548945 | 2 | 217665788 | T | C | 0.96 | 1.70E-06 |
| rs1050816 | 2 | 220358198 | T | C | 0.97 | 0.00016 |
| rs13003198 | 2 | 234257105 | T | C | 0.98 | 0.0067 |
| rs795009 | 3 | 12208671 | T | G | 0.97 | 0.0024 |
| rs6778731 | 3 | 13947504 | T | C | 1.02 | 0.01 |
| rs6779998 | 3 | 30749965 | A | G | 1.03 | 8.20E-05 |
| rs11914389 | 3 | 38527215 | T | C | 0.98 | 0.0026 |
| rs7651407 | 3 | 48443816 | T | C | 0.97 | 0.001 |
| rs4625 | 3 | 49572140 | A | G | 1.02 | 0.032 |
| rs3774726 | 3 | 63974477 | T | C | 1.03 | 4.30E-05 |
| rs2289746 | 3 | 105455955 | T | C | 1.02 | 0.046 |
| rs9868185 | 3 | 121657593 | A | G | 0.97 | 0.00074 |
| rs10934754 | 3 | 125906237 | T | C | 0.97 | 0.00016 |
| rs7624084 | 3 | 141093285 | T | C | 0.97 | 1.60E-05 |
| rs1397764 | 3 | 141750810 | A | G | 0.97 | 9.00E-04 |
| rs56065557 | 3 | 185354216 | C | G | 1.02 | 0.015 |
| rs11919484 | 3 | 186432839 | T | G | 1.03 | 4.60E-05 |
| rs9823161 | 3 | 193811168 | A | G | 0.98 | 0.0097 |
| rs16874073 | 4 | 23743962 | T | C | 1.08 | 3.90E-05 |
| rs4864890 | 4 | 52686513 | T | C | 1.02 | 0.022 |
| rs223471 | 4 | 103698786 | C | G | 0.98 | 0.0022 |
| rs71606723 | 4 | 115498457 | A | T | 0.97 | 0.0033 |
| rs13159523 | 5 | 676962 | A | G | 1.03 | 0.00029 |
| rs13157326 | 5 | 34504277 | A | G | 1.04 | 2.60E-06 |
| rs1362800 | 5 | 39378115 | T | C | 1.06 | 4.60E-11 |
| rs495237 | 5 | 39950266 | T | G | 0.97 | 0.00061 |
| rs11746506 | 5 | 44812566 | T | C | 0.98 | 0.0062 |
| rs12520984 | 5 | 52787358 | C | G | 0.98 | 0.015 |
| rs79760705 | 5 | 53298716 | T | G | 0.95 | 0.00091 |
| rs72759880 | 5 | 67750213 | T | G | 1.05 | 0.00039 |
| rs2010352 | 5 | 68656327 | A | G | 1.02 | 0.01 |
| rs12163971 | 5 | 132226669 | A | C | 1.04 | 0.00098 |
| rs11743174 | 5 | 148524820 | T | C | 0.97 | 0.00068 |
| rs3812036 | 5 | 176813404 | T | C | 1.07 | 1.20E-12 |
| rs3765502 | 6 | 24354045 | T | C | 0.98 | 0.03 |
| rs144100226 | 6 | 34180297 | T | C | 0.92 | 0.00036 |
| rs13200335 | 6 | 41690823 | A | C | 0.98 | 0.0046 |
| rs77915916 | 6 | 43287722 | A | T | 0.95 | 0.00077 |
| rs881858 | 6 | 43806609 | A | G | 1.06 | 6.40E-11 |
| rs720989 | 6 | 44765535 | T | G | 0.97 | 0.00029 |
| rs12212034 | 6 | 51492862 | T | C | 1.02 | 0.0049 |
| rs6458868 | 6 | 52630153 | T | C | 1.02 | 0.0048 |
| rs3925003 | 6 | 55422618 | T | C | 1.03 | 3.70E-05 |
| rs72912510 | 6 | 90118764 | A | G | 1.05 | 2.00E-05 |
| rs1857859 | 6 | 100894587 | A | G | 0.98 | 0.005 |
| rs7740107 | 6 | 130374461 | A | T | 0.95 | 1.30E-06 |
| rs9375818 | 6 | 131882078 | A | G | 1.04 | 2.20E-06 |
| rs3822939 | 6 | 133849789 | A | G | 1.03 | 0.00017 |
| rs12207180 | 6 | 160633107 | A | T | 1.09 | 1.10E-10 |
| rs62435145 | 7 | 1286567 | T | G | 1.07 | 2.90E-12 |
| rs6968554 | 7 | 17287106 | A | G | 1.02 | 0.014 |
| rs3750081 | 7 | 32930876 | T | G | 1.03 | 0.00029 |
| rs55773927 | 7 | 65337902 | T | C | 0.98 | 0.015 |
| rs801193 | 7 | 66030612 | T | G | 1.02 | 0.047 |
| rs41301394 | 7 | 75612803 | T | C | 0.97 | 0.00014 |
| rs6973656 | 7 | 77422583 | A | G | 0.98 | 0.0028 |
| rs62491533 | 7 | 129564134 | T | C | 1.03 | 0.0031 |
| rs10254101 | 7 | 151415536 | T | C | 1.11 | 1.20E-25 |
| rs12671694 | 7 | 155665959 | T | C | 0.97 | 0.00085 |
| rs868822 | 7 | 156252939 | T | G | 0.95 | 2.60E-08 |
| rs2980423 | 8 | 8142575 | T | C | 1.03 | 0.002 |
| rs1533059 | 8 | 8684953 | A | G | 0.98 | 0.01 |
| rs35353426 | 8 | 9297246 | T | C | 1.03 | 0.0023 |
| rs7832708 | 8 | 10190040 | T | C | 0.98 | 0.015 |
| rs11783418 | 8 | 10841858 | A | G | 1.02 | 0.036 |
| rs10098664 | 8 | 11417493 | T | C | 1.02 | 0.018 |
| rs34861762 | 8 | 23748420 | T | C | 1.04 | 1.20E-07 |
| rs10102889 | 8 | 32435620 | C | G | 1.04 | 0.005 |
| rs2976178 | 8 | 87332552 | C | G | 1.03 | 0.00036 |
| rs2954017 | 8 | 126476873 | T | C | 0.98 | 0.047 |
| rs13287724 | 9 | 33169034 | A | T | 1.05 | 0.00055 |
| rs544169 | 9 | 33956791 | A | G | 0.98 | 0.0094 |
| rs2039424 | 9 | 71432174 | A | G | 0.96 | 5.40E-08 |
| rs1321917 | 9 | 119324929 | C | G | 1.02 | 0.037 |
| rs7024579 | 9 | 139100413 | T | C | 0.98 | 0.034 |
| rs80282103 | 10 | 899071 | A | T | 0.91 | 9.90E-11 |
| rs6481598 | 10 | 29781798 | C | G | 0.96 | 3.50E-05 |
| rs7072591 | 10 | 35150364 | A | G | 0.98 | 0.024 |
| rs10821905 | 10 | 52646093 | A | G | 0.97 | 0.0073 |
| rs7475348 | 10 | 69965177 | T | C | 0.96 | 7.20E-06 |
| rs12240572 | 10 | 75016365 | A | T | 1.04 | 0.0025 |
| rs816850 | 10 | 79252446 | C | G | 1.02 | 0.04 |
| rs7095954 | 10 | 82209232 | A | T | 1.02 | 0.0085 |
| rs2068888 | 10 | 94839642 | A | G | 1.02 | 0.0019 |
| rs4918943 | 10 | 97278922 | A | G | 1.03 | 0.00045 |
| rs284859 | 10 | 104573017 | T | G | 0.97 | 0.0078 |
| rs1536225 | 10 | 105202318 | T | G | 1.02 | 0.014 |
| rs1055256 | 10 | 126446592 | A | G | 0.97 | 2.00E-04 |
| rs11564722 | 11 | 2178330 | T | C | 0.98 | 0.031 |
| rs63934 | 11 | 2789062 | A | G | 0.97 | 0.002 |
| rs963837 | 11 | 30749090 | T | C | 1.08 | 1.60E-21 |
| rs6484504 | 11 | 31424823 | T | C | 1.03 | 0.00068 |
| rs61897431 | 11 | 47427667 | T | C | 0.96 | 3.20E-07 |
| rs7127946 | 11 | 48250675 | T | C | 0.96 | 6.20E-08 |
| rs2727040 | 11 | 49057603 | T | C | 1.06 | 7.80E-07 |
| rs1813937 | 11 | 50468801 | T | C | 0.95 | 1.30E-07 |
| rs948493 | 11 | 65552154 | T | C | 1.06 | 7.50E-12 |
| rs11237450 | 11 | 78023356 | A | C | 0.96 | 3.80E-05 |
| rs6589750 | 11 | 119326726 | A | G | 0.97 | 0.0014 |
| rs10790452 | 11 | 121584931 | T | C | 0.96 | 3.10E-05 |
| rs632887 | 12 | 3392351 | A | G | 0.97 | 0.0011 |
| rs4238020 | 12 | 4616642 | T | C | 0.96 | 0.0035 |
| rs117113238 | 12 | 12209203 | A | G | 0.96 | 0.021 |
| rs10846157 | 12 | 15325031 | A | C | 1.05 | 2.10E-06 |
| rs2634675 | 12 | 48740855 | A | G | 0.97 | 0.0013 |
| rs12313306 | 12 | 57751854 | T | C | 0.96 | 5.30E-05 |
| rs41284816 | 13 | 50655989 | T | G | 1.09 | 0.0083 |
| rs6574652 | 14 | 81870100 | T | C | 1.02 | 0.019 |
| rs1028455 | 14 | 88829975 | A | T | 0.97 | 0.00076 |
| rs61993680 | 14 | 100752644 | A | C | 1.02 | 0.012 |
| rs12913015 | 15 | 39305443 | T | C | 0.96 | 3.30E-06 |
| rs6492982 | 15 | 41399951 | T | C | 1.03 | 0.00036 |
| rs1145077 | 15 | 45683795 | T | G | 1.07 | 2.50E-16 |
| rs690428 | 15 | 53950578 | A | C | 1.07 | 9.80E-15 |
| rs956006 | 15 | 62808539 | T | C | 0.98 | 0.0078 |
| rs11071738 | 15 | 63580155 | T | C | 1.03 | 0.00014 |
| rs351237 | 15 | 74477239 | A | G | 1.02 | 0.021 |
| rs4886696 | 15 | 75664570 | A | T | 1.02 | 0.02 |
| rs438339 | 16 | 2003425 | T | C | 0.96 | 0.029 |
| rs1635404 | 16 | 3747042 | T | G | 1.04 | 0.00016 |
| rs9932625 | 16 | 51735746 | A | G | 1.03 | 0.00012 |
| rs7185391 | 16 | 68323115 | T | G | 1.03 | 0.00084 |
| rs62050038 | 16 | 69802865 | A | T | 0.96 | 0.00031 |
| rs62053077 | 16 | 71643669 | T | G | 1.03 | 0.0016 |
| rs28581385 | 16 | 79942679 | A | T | 1.03 | 0.0098 |
| rs28735420 | 17 | 12139964 | T | G | 0.95 | 0.00061 |
| rs9891340 | 17 | 17543846 | T | C | 0.98 | 0.0029 |
| rs2440165 | 17 | 19428719 | T | C | 0.95 | 2.70E-07 |
| rs2411192 | 17 | 34882998 | A | T | 1.05 | 4.20E-09 |
| rs4794813 | 17 | 37670994 | A | T | 0.96 | 7.60E-05 |
| rs227731 | 17 | 54773238 | T | G | 0.97 | 0.00053 |
| rs9903801 | 17 | 58915261 | C | G | 0.97 | 0.0013 |
| rs9895661 | 17 | 59456589 | T | C | 0.95 | 4.70E-07 |
| rs8866 | 17 | 65373979 | C | G | 1.02 | 0.031 |
| rs1719934 | 18 | 5585158 | A | G | 0.96 | 4.20E-06 |
| rs16942751 | 18 | 24393213 | A | C | 1.08 | 2.10E-10 |
| rs4940525 | 18 | 59354616 | T | C | 0.98 | 0.0068 |
| rs2974751 | 19 | 13053034 | A | C | 0.98 | 0.013 |
| rs8101667 | 19 | 33402419 | T | C | 0.96 | 1.70E-06 |
| rs7251730 | 19 | 36997147 | T | C | 0.98 | 0.048 |
| rs78241494 | 19 | 37649748 | T | C | 1.03 | 0.0084 |
| rs113445505 | 19 | 38157969 | T | C | 0.97 | 0.0033 |
| rs281380 | 19 | 49214470 | T | C | 1.03 | 0.00034 |
| rs34647824 | 19 | 50138143 | A | C | 1.04 | 3.30E-05 |
| rs1041606 | 20 | 14677788 | T | C | 1.02 | 0.042 |
| rs6087579 | 20 | 32985155 | A | G | 1.03 | 0.0013 |
| rs2273684 | 20 | 33529766 | T | G | 0.98 | 0.0022 |
| rs17216707 | 20 | 52732362 | T | C | 1.05 | 1.30E-05 |
| rs2235826 | 20 | 56143169 | A | T | 1.03 | 0.0013 |
| rs1407040 | 20 | 57472174 | T | C | 0.98 | 0.027 |
| rs35636653 | 20 | 60858758 | T | C | 0.97 | 0.00034 |
| rs72629024 | 20 | 62152519 | C | G | 0.96 | 0.0016 |
| rs4408777 | 20 | 62706105 | A | G | 1.04 | 2.30E-06 |
| rs2834317 | 21 | 35356706 | A | G | 1.05 | 0.00028 |
| rs131263 | 22 | 30133045 | T | C | 0.97 | 0.0041 |
| rs80576 | 22 | 36539804 | A | G | 1.04 | 0.00053 |
| rs4820324 | 22 | 38599857 | C | G | 1.03 | 0.001 |
| rs738527 | 22 | 43112961 | T | C | 0.96 | 3.80E-05 |

SNP: single nucleotide polymorphism; CHR Chromosome;

Supplementary File 1-2. Baseline characteristics of 65,069 UK Biobank participants according to the working conditions risk score.

|  | Working conditions risk score | | | | | | |
| --- | --- | --- | --- | --- | --- | --- | --- |
| Baseline characteristics | Overall | 0 | 1 | 2 | 3 | 4 | p for trend |
|  | 65069 | 19383 | 27163 | 15305 | 2829 | 389 |  |
| Females (%) | 29819 (45.8) | 7782 (40.1) | 11715 (43.1) | 8218 (53.7) | 1861 (65.8) | 243 (62.5) | <0.001 |
| Age (year) | 65.6 (6.8) | 65.1 (7.0) | 65.9 (6.8) | 65.9 (6.7) | 65.2 (6.7) | 63.5 (6.4) | <0.001 |
| Ethnic.White (%) | 59851 (92.0) | 17630 (91.0) | 25071 (92.3) | 14176 (92.6) | 2622 (92.7) | 352 (90.5) | <0.001 |
| Working time (hour/week) | 35.3 (12.5) | 34.6 (12.7) | 35.0 (12.4) | 36.1 (12.3) | 38.1 (12.5) | 38.9 (11.8) | <0.001 |
| Smoking (%) | 4886 ( 7.5) | 1093 ( 5.6) | 1918 ( 7.1) | 1460 ( 9.5) | 355 (12.5) | 60 (15.4) | <0.001 |
| Alcohol_frequency (%) |  |  |  |  |  |  | <0.001 |
| Daily or almost daily | 14125 (21.7) | 4168 (21.5) | 6159 (22.7) | 3219 (21.0) | 525 (18.6) | 54 (13.9) |  |
| 3-4 times a week | 17836 (27.4) | 5535 (28.6) | 7478 (27.5) | 4062 (26.5) | 684 (24.2) | 77 (19.8) |  |
| 1-2 times a week | 17257 (26.5) | 5026 (25.9) | 7082 (26.1) | 4209 (27.5) | 827 (29.2) | 113 (29.0) |  |
| 1-3 times a month | 7368 (11.3) | 2190 (11.3) | 3025 (11.1) | 1739 (11.4) | 354 (12.5) | 60 (15.4) |  |
| Special occasions only | 5451 ( 8.4) | 1540 ( 7.9) | 2208 ( 8.1) | 1369 ( 8.9) | 289 (10.2) | 45 (11.6) |  |
| Never | 3032 ( 4.7) | 924 ( 4.8) | 1211 ( 4.5) | 707 ( 4.6) | 150 ( 5.3) | 40 (10.3) |  |
| Activity_MET (%) |  |  |  |  |  |  | 0.766 |
| Low | 15572 (23.9) | 4658 (24.0) | 6454 (23.8) | 3686 (24.1) | 674 (23.8) | 100 (25.7) |  |
| Medium | 18561 (28.5) | 5519 (28.5) | 7780 (28.6) | 4387 (28.7) | 774 (27.4) | 101 (26.0) |  |
| High | 30936 (47.5) | 9206 (47.5) | 12929 (47.6) | 7232 (47.3) | 1381 (48.8) | 188 (48.3) |  |
| GRS_CKD (%) |  |  |  |  |  |  | 0.438 |
| Low | 21139 (32.5) | 6276 (32.4) | 8780 (32.3) | 4992 (32.6) | 965 (34.1) | 126 (32.4) |  |
| Medium | 21525 (33.1) | 6434 (33.2) | 9038 (33.3) | 5001 (32.7) | 934 (33.0) | 118 (30.3) |  |
| High | 22405 (34.4) | 6673 (34.4) | 9345 (34.4) | 5312 (34.7) | 930 (32.9) | 145 (37.3) |  |
| TDI (mean (SD)) | -1.7 (2.8) | -1.8 (2.8) | -1.7 (2.8) | -1.6 (2.8) | -1.2 (3.0) | -1.0 (2.8) | <0.001 |
| College (%) | 33548 (51.6) | 12628 (65.1) | 14527 (53.5) | 5916 (38.7) | 432 (15.3) | 45 (11.6) | <0.001 |
| Healthy diet (%) | 53347 (82.0) | 16149 (83.3) | 22414 (82.5) | 12312 (80.4) | 2181 (77.1) | 291 (74.8) | <0.001 |
| DM (%) | 2951 ( 4.5) | 685 ( 3.5) | 1165 ( 4.3) | 889 ( 5.8) | 185 ( 6.5) | 27 ( 6.9) | <0.001 |
| CVD (%) | 1868 ( 2.9) | 409 ( 2.1) | 775 ( 2.9) | 566 ( 3.7) | 101 ( 3.6) | 17 ( 4.4) | <0.001 |
| Hypertension (%) | 12085 (18.6) | 3049 (15.7) | 5074 (18.7) | 3265 (21.3) | 612 (21.6) | 85 (21.9) | <0.001 |
| eGFR (ml/min/1.73 m^2^) | 94.5 (15.2) | 94.6 (15.2) | 94.3 (15.1) | 94.7 (15.2) | 95.8 (15.7) | 97.0 (16.1) | <0.001 |
| BMI (kg/m^2^) | 26.7 (4.5) | 26.0 (4.3) | 26.6 (4.5) | 27.3 (4.7) | 27.8 (4.7) | 28.3 (4.8) | <0.001 |
| SBP (mmHg) | 134.2 (17.3) | 132.8 (17.2) | 134.2 (17.3) | 135.7 (17.3) | 136.7 (16.8) | 134.4 (15.9) | <0.001 |
| DBP (mmHg) | 81.8 (10.0) | 81.0 (9.9) | 81.7 (9.9) | 82.6 (10.0) | 83.2 (9.8) | 82.8 (10.0) | <0.001 |
| FBG (mmol/L) | 5.0 (1.0) | 5.0 (0.9) | 5.0 (1.0) | 5.0 (1.1) | 5.1 (1.1) | 5.0 (0.8) | <0.001 |
| TC (mmol/L) | 5.7 (1.1) | 5.7 (1.1) | 5.7 (1.1) | 5.7 (1.1) | 5.7 (1.1) | 5.6 (1.0) | 0.002 |
| TG (mmol/L) | 1.6 (1.0) | 1.5 (0.9) | 1.6 (1.0) | 1.7 (1.1) | 1.8 (1.0) | 1.8 (1.1) | <0.001 |
| HDL (mmol/L) | 1.5 (0.4) | 1.5 (0.4) | 1.5 (0.4) | 1.4 (0.4) | 1.4 (0.3) | 1.3 (0.3) | <0.001 |
| LDL (mmol/L) | 3.6 (0.8) | 3.5 (0.8) | 3.6 (0.8) | 3.6 (0.8) | 3.6 (0.8) | 3.5 (0.8) | <0.001 |
| HbA1c (mmol/mol) | 5.3 (0.5) | 5.3 (0.5) | 5.3 (0.5) | 5.4 (0.5) | 5.4 (0.6) | 5.4 (0.4) | <0.001 |

Values are mean±SD or n (%)

DM: diabetes mellitus, CVD: cardiovascular disease, TDI: townsend deprivation index, MET: metabolic equivalent task, GRS_CKD: genetic risk score of chronic kidney disease, SBP: systolic blood pressure, DBP: diastolic blood pressure, BMI: body mass index, WC: waist circumference, FPG: fasting plasma glucose, HbA1c: glycated hemoglobin, TG: total triglyceride, TC: total cholesterol, HDL-C: high densitylipoprotein cholesterol, LDL-C: low density lipoprotein cholesterol, eGFR: estimated glomerular filtration rate.

## Supplementary File 1-3. Multivariable-adjusted HRs (95%CIs) for incident CKD by risky working conditions among 65,069 participants.

|  | HR (95% CI) | p-value |
| --- | --- | --- |
| **Heat** |  |  |
| Never/rarely | 1.00(reference) | - |
| Sometimes | 1.10( 0.98 , 1.24) | 0.091 |
| often | 1.16(0.91 , 1.47) | 0.232 |
| **Secondhand smoke** |  |  |
| Never/rarely | 1.00(reference) | - |
| Sometimes | 1.10(0.97 , 1.24) | 0.129 |
| often | 1.22(1.04 , 1.44) | 0.018 |
| **Heavy workloads** |  |  |
| Never/rarely | 1.00(reference) | - |
| Sometimes | 1.09(0.95 , 1.26) | 0.216 |
| Usually | 1.17(0.90 , 1.50) | 0.239 |
| Always | 1.41(1.07 , 1.87) | 0.016 |
| **Shift work** |  |  |
| Never/rarely | 1.00(reference) | - |
| Sometimes | 1.07(0.85 , 1.33) | 0.577 |
| Usually | 1.37(0.93 , 2.02) | 0.117 |
| Always | 1.34(1.07 , 1.67) | 0.010 |

Multivariable-adjusted HRs: Age (continuous), Sex (male/female), Ethnic (Whiter/other)，Working time(continuous), Activity_MET (low/median/high), The Townsend Deprivation Index (continuous), Smoke status (yes/no), Alcohol consumption (frequence1-6), TG(continuous), Body mass index (continuous), Hypertension (yes/no), DM (yes/no), eGFR(continuous).

Supplementary File 1-4. Multivariable-adjusted HRs (95%CIs) for incident CKD by different weighted working conditions risk score.

|  | Model1 | | Model2 | | Model3 | |
| --- | --- | --- | --- | --- | --- | --- |
| Weighted score* | HR (95% CI) | p-value | HR (95% CI) | p-value | HR (95% CI) | p-value |
| 0 | 1.00(reference) | - | 1.00(reference) | - | 1.00(reference) | - |
| 0~<=1 | 1.22( 1.05 , 1.42) | 0.008 | 1.21( 1.05 , 1.41) | 0.010 | 1.16( 1.00 , 1.34) | 0.052 |
| 1~<=2 | 1.46( 1.24 , 1.71) | <0.001 | 1.42( 1.21 , 1.66) | <0.001 | 1.30( 1.10 , 1.52) | 0.002 |
| 2~<=3 | 1.64( 1.29 , 2.08) | <0.001 | 1.53( 1.20 , 1.94) | <0.001 | 1.37( 1.08 , 1.74) | 0.010 |
| 3~<=4 | 2.08( 1.36 , 3.18) | 0.001 | 1.86( 1.21 , 2.85) | 0.005 | 1.71( 1.12 , 2.62) | 0.014 |

Model1. Age (continuous), Sex (male/female), Ethnic (Whiter/other), Working time(continuous).

Model2. model1+Activity_MET (low/median/high), The Townsend Deprivation Index (continuous), Alcohol consumption (frequence1-6), Smoke status (yes/no).

Model3. model2+TG(continuous), Body mass index (continuous), Hypertension (yes/no), DM (yes/no), eGFR(continuous).

* We constructed a weighted working conditions risk score based on the 4 work factors by using the equation: weighted work score= (β1×factor1 +β2 ×factor 2 +…+β4×factor 4) × (4/sum of the β coefficients). This weighted score also ranges from 0 to 4 points but considers magnitudes of the adjusted relative risk for each factor in each work pattern as a combination of 4 factors.

Supplementary File 1-5. Associations between the categorized working conditions risk score and incident CKD after further adjusting for CKD-GRS, education and health diet.

|  | Model3 | | Model3+CKD-GRS | | Model3+CKD-GRS+education+health diet | |
| --- | --- | --- | --- | --- | --- | --- |
|  | HR (95% CI) | p-value | HR (95% CI) | p-value | HR (95% CI) | p-value |
| Healthy conditions | 1.00(reference) | - | 1.00(reference) | - | 1.00(reference) | - |
| Intermediate conditions | 1.18(1.04 , 1.35) | 0.012 | 1.18(1.04 , 1.35) | 0.012 | 1.15(1.01 , 1.31) | 0.038 |
| Poor conditions | 1.50(1.19 , 1.91) | <0.001 | 1.51(1.19 , 1.91) | <0.001 | 1.39(1.09 , 1.77) | 0.008 |

Model3: Age (continuous), Sex (male/female), Ethnic (Whiter/other)，Working time(continuous), Activity_MET (low/median/high), The Townsend Deprivation Index (continuous), Smoke status (yes/no), Alcohol consumption (frequence1-6), TG(continuous), Body mass index (continuous), Hypertension (yes/no), DM (yes/no), eGFR(continuous).

Supplementary File 1-6 . Associations betweenthe categorized working conditions risk score and incident CKD after further excluding incident cases occurred in the first 2 years of follow-up.

|  | Model1 |  | Model2 |  | Model3 |  |
| --- | --- | --- | --- | --- | --- | --- |
|  | HR (95% CI) | p-value | HR (95% CI) | p-value | HR (95% CI) | p-value |
| Healthy conditions | 1.00(reference) | - | 1.00(reference) | - | 1.00(reference) | - |
| Intermediate conditions | 1.27(1.11 , 1.46) | 0.001 | 1.25(1.09 , 1.43) | 0.002 | 1.17(1.02 , 1.34) | 0.026 |
| Poor conditions | 1.82(1.42 , 2.34) | <0.001 | 1.679(1.31 , 2.15) | <0.001 | 1.51(1.17 , 1.93) | 0.001 |

Model1. Age (continuous), Sex (male/female), Ethnic (Whiter/other), Working time(continuous).

Model2. model1+Activity_MET (low/median/high), The Townsend Deprivation Index (continuous), Alcohol consumption (frequence1-6), Smoke status (yes/no).

Model3. model2+TG(continuous), Body mass index (continuous), Hypertension (yes/no), DM (yes/no), eGFR(continuous).
